# Understanding the impact of the unique stressors of National Guard service: the National Guard Occupational Demands Model

**DOI:** 10.3389/fpsyg.2026.1958672

**Published:** 2026-09-18

**Authors:** Sara Kintzle, Nicholas Barr, Justin Jaesung Lee, Eva Alday, Carl A. Castro

**Affiliations:** 1 Suzanne Dworak-Peck School of Social Work, University of Southern California, Los Angeles, CA, United States; 2 The University of Utah College of Social Work, Salt Lake City, UT, United States

**Keywords:** health, military, National Guard, occupational demands, stressors, wellbeing

## Abstract

The National Guard (NG) is a vital component of the U.S. Armed Forces, balancing federal and state responsibilities that serve and protect the Nation. Those who serve in the NG have a unique and understudied role within the military. As part-time service members, NG must balance the functional demands of their civilian roles and responsibilities with their military roles, and obligations. Despite their distinct service experience, NG research, intervention and policy often fail to recognize important factors that distinguish these service members from their active-duty military peers, as well as the unique demands and stressors of NG service. This paper presents a conceptual framework for understanding the occupational demands of NG service. The National Guard Occupational Demands Model (NGODM) describes the demands associated with NG service, the impact of those demands on NG organizational goals and service member wellbeing and variables that may impact the relationship between demands and outcomes.

## Introduction

The National Guard (NG) is a unique component of the military within the U.S. Armed Forces. Comprised of the Army National Guard and the Air Force Air National Guard, the NG is composed of service members who primarily serve in part-time roles, providing support and protection for our States and domestic communities (i.e., responding in times of crisis), as well as reinforcing the active-duty forces of the U.S. military in defense of the nation. This dual role means NG are standing by to be activated by the President of the United States or mobilized by state governors (United States National Guard, n.d.). It also means NG service members must navigate the distinctive experience of simultaneously balancing the functional demands of their civilian roles and responsibilities with their military roles and obligations.

Although NG service members sign contracts to perform inactive duty training (IDT; also referred to as drill) for a weekend each month and 2 weeks of annual training (AT) (Binkin and Kaufmann, 2010), it is important to appreciate that NG obligations often go well beyond these baseline commitments. Expanded or increased drill, mandatory extra trainings, and mobilizations at the state or federal level are common demands for those serving in the NG. Over the past 25 years, the U.S. has seen the role and use of the NG dramatically shift and intensify (Jones, 2012; Mahoney, 2005; Palme et al., 2025; Radi et al., 2022). NG units were activated and deployed at unprecedented numbers during the global war on terrorism starting in the early 2000s. NG performed critical combat missions in Iraq and Afghanistan and assumed mission leads in ongoing peacekeeping missions in Bosnia and Herzegovina, and Kosovo. Despite the drawdown of those conflicts, NG continue to serve and deploy as key components of the total forces overseas (U.S. Department of Defense, 2021). Additionally, the past 10 years have seen domestic shifts and increases in the use of NG. This has included responding to natural disasters, civil unrest, COVID-19, and providing support to local, state and federal law enforcement in response to crime, drug enforcement, election security demands, and immigration border control. The dual civilian/military identity inherent in NG service, the shifting roles of the NG, and increased domestic and federal activations create unique demands and occupational stressors for these service members that have yet to be robustly defined or investigated.

Literature on the experiences of NG primarily centers on Iraq and Afghanistan deployments, mobilizations in response to COVID-19, and the impact of deployment on NG members mental and behavioral health outcomes. This body of work suggests that NG members who have been deployed are at increased risk of mental health issues such as PTSD, depression, insomnia and anxiety, like their active-duty counterparts (Riviere et al., 2024; Sampson et al., 2021). Conversely, studies examining never-deployed NG populations have found that deployment had less impact on mental and behavioral health outcomes than factors such as motivations for joining the service, resilience, unit support, and psychological hardiness (Hoopsick et al., 2020, 2021a,^2025^; Griffith, 2022). Research has also shown strong positive associations between experiences of financial struggles in NG populations and anger and PTSD symptom severity; NG service members who reported higher levels of PTSD symptom severity, or reported experiencing sudden unemployment, homelessness, or financial struggles, had higher rates of problematic anger (Worthen et al., 2014; Adler et al., 2022).

Some research suggests that NG service members are at greater risk of adverse mental health outcomes, such as aggression, post deployment PTSD, and problematic alcohol and substance use compared to active-duty service members (Hoopsick et al., 2018; Ursano et al., 2018; Thomas et al., 2010). Further, robust literature has shown high risk of problematic alcohol and substance use in NG populations. This increased risk has been linked to factors such as feelings associated with non-deployment (Hoopsick et al., 2021b), lack of pre-deployment preparation (Riviere et al., 2024), military identity (Vest et al., 2023) and sleep issues (Vest et al., 2022).

There are several factors unique to NG service that contribute to the heightened risks and stressors these service members experience. The added responsibility of navigating their civilian employment and responsibilities in conjunction with service obligations, military training, and deployments, can create a significant stress burden. Further, NG members experience abrupt transitions back to civilian life, unexpected deployment schedules, and sometimes fragmented access to physical and behavioral health services due to limited or discontinued coverage of military medical care upon return to civilian status (Hardison and Ginsberg, 2024). NG members enrolled in higher education programs can experience challenges due to limited access to educational benefits relative to active-duty service members, as well as difficulties completing educational programs due to increased mobilizations, unpredictable schedules and added transitional stressors (Brannan, 2023; Vest et al., 2020). A recent RAND report investigating stressors of NG service highlighted additional challenges NG members experience, which include interpersonal disconnectedness, increased personnel and operational tempos, financial stress related to differing duty statuses, gaps in healthcare coverage, and challenges in access to childcare (Hardison and Ginsberg, 2024).

While there is limited empirical literature investigating the experiences, health, and wellbeing of individuals who serve in the NG, it has many limitations. Investigatory approaches with NG populations have been fragmented and unsystematic, lacking the cohesion, integration and comprehensiveness needed to form a body of literature that can be generalized to support the broader NG. In addition, much of the NG research is limited by small samples, lack of replication and generalizability of results, and limited theoretical grounding. NG research appears to be often driven by a narrow topic focus at the expense of a comprehensive framework for understanding the interrelated domains of wellbeing uniquely influenced by population demand characteristics. In other words, researchers have used the NG population to study a topic in their sphere of expertise or interest as opposed to building knowledge within the NG experience. Lastly, there are significant gaps in the literature on NG, including some of the most important topics related to NG service, the wellbeing of NG members, and the organization as a whole. Examples of these include, but are not limited to, service demands and stressors, retention, leadership, physical and mental wellbeing outside of deployment, wellbeing factors outside of mental health, civilian employment challenges, managing dual roles, connectedness and community, care access and utilization, the experience of never deployed or activated NG and the experience and stressors of spouses and families.

## Occupational stress theories and military service

The military has historically applied a number of different psychological or social theories related to occupational stress to identify and describe physical, mental, and behavioral outcomes of service, risk and protective factors associated with those outcomes, and points of intervention that may bolster resilience, minimize risk, and facilitate recovery (see for example, Bliese and Castro, 2000). Relatively recently, there has been a shift toward the development of theories and frameworks specifically designed to incorporate the cultural aspects of military service. The most prominent of these theoretical models is the MOMHM developed by Adler and Castro (2013). The MOMHM presents a framework for understanding the occupational demands associated with military service, how those demands impact mental health, and individual and organizational resources that may mitigate the impact of service demands. The model’s key domains include background, demands, outcomes, and goals, while personal and occupational resources function as potential moderating factors. The MOMHM built on and expanded the Soldier Adaptation Model, a military stressor-strain model metatheory of occupational stress influenced by theories such as demands-control theory (Karasek, 1979), stress efficacy theory (Jex and Bliese, 1999), and the triangle model of responsibility (Britt, 1999). Such models provided an important foundation for thinking about how the military organization, leaders and service members can protect against the potential negative impacts of high-risk occupations like military service.

The MOMHM has been applied to guide research, intervention, training, and resources to protect service members against the known demands and stressors of military service, including combat, deployments and training exercises. While the model has been critical in providing the conceptual framework for understanding the occupational demands of active-duty service members, it has limitations when applied to NG service members. As we have shown, NG are unique, with service demands and structural challenges that differ substantially from those of their active-duty counterparts. It is non-controversial to state that maintaining a mission ready part-time force requires different resources and strategies than the full-time force. Yet, the NG literature lacks a theoretical foundation that delineates key components of this distinct service experience to better guide research, training and intervention to mitigate the demands and risks of NG service.

## The National Guard Occupational Demands Model (NGODM)

The limitations and gaps in the NG research landscape, and the inherent duality and evolving nature of modern era NG service demands, call for a more robust theoretical account of the NG experience to better support NG service members and NG readiness. The objective of this paper is to address this gap by introducing a comprehensive conceptual framework for understanding the unique demands of NG service and associations between these demands and individual and organizational outcomes. We aim to (1) synthesize the fragmented empirical literature on NG stressors and domains of wellbeing to produce a coherent set of constructs for investigation; (2) extend the previously developed Military Occupational Mental Health Model (MOHM; Adler and Castro, 2013) to account for the dual civilian-military role and unique structural demands that characterize NG service; and (3) advance a theoretical model to generate testable propositions and guide future research, clinical intervention, and policy aimed at supporting NG members. The model was developed through a narrative synthesis of existing theoretical and empirical literature on occupational stress, military mental health and wellbeing, and NG specific research, informed by the authors’ clinical and research expertise with this population.

Built upon the MOMHM, the National Guard Occupational Demands Model (NGODM; see Figures 1, 2) describes the goals of NG service, the outcomes that lead to success in those goals, background and occupational demands that impact outcomes, and the personal and occupational resources that may moderate the relationship between demands and outcomes. Each section of the model is displayed, described and discussed in detail below. Examples of operationalized variables are also provided under each model construct. It is important to note that the model is not inclusive of every potential construct or example of the NG experience, but those the authors have identified as key areas to inform conceptualization and empirical investigation of NG occupational stress domains and their associations. It is our hope that the NGODM can serve as a starting point for advancing our thinking and understanding about NG service; we expect the model will evolve as researchers begin to test this framework.

**FIGURE 1:**
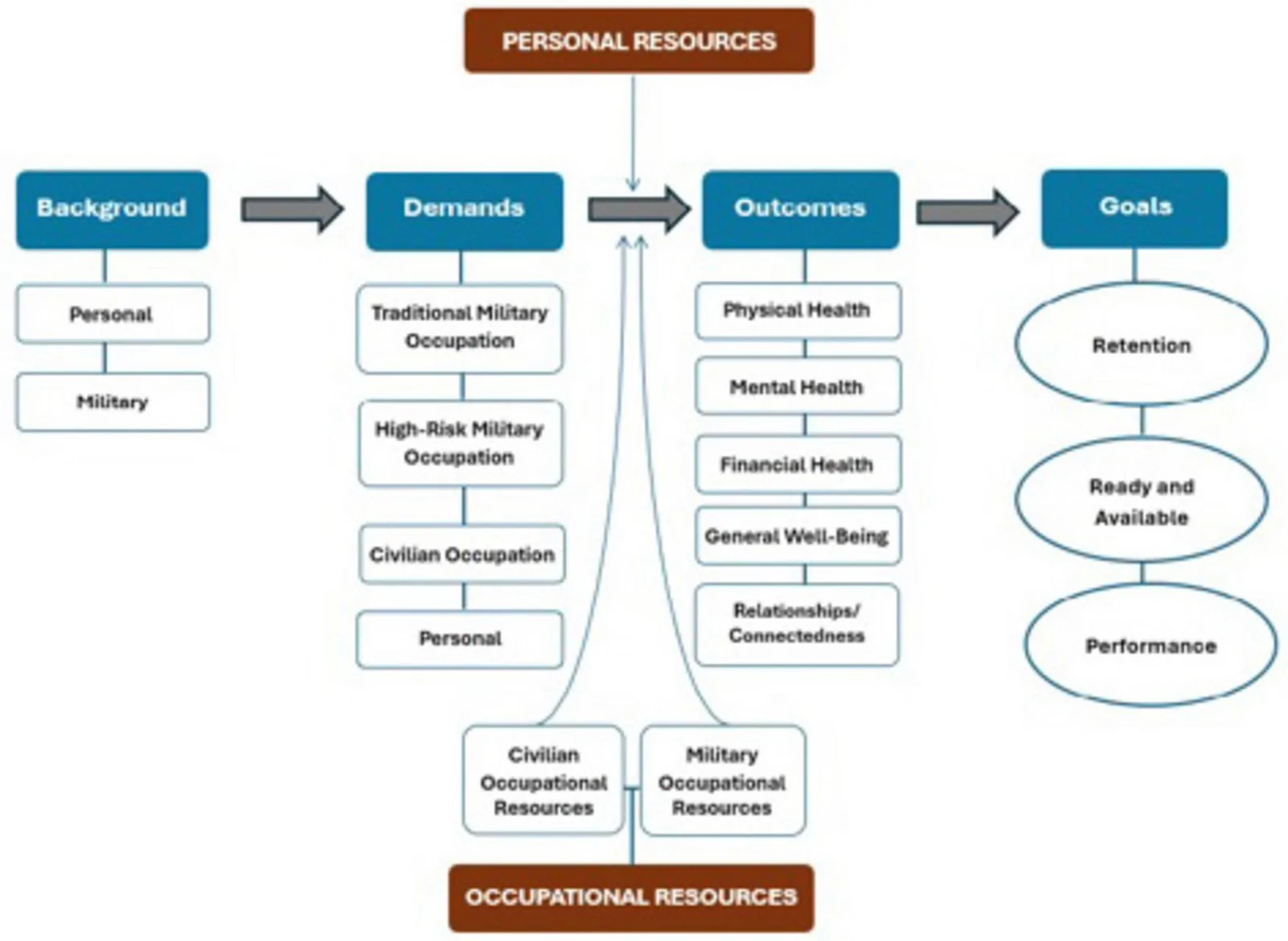
The National Guard Occupational Demands Model (NGODM).

**FIGURE 2:**
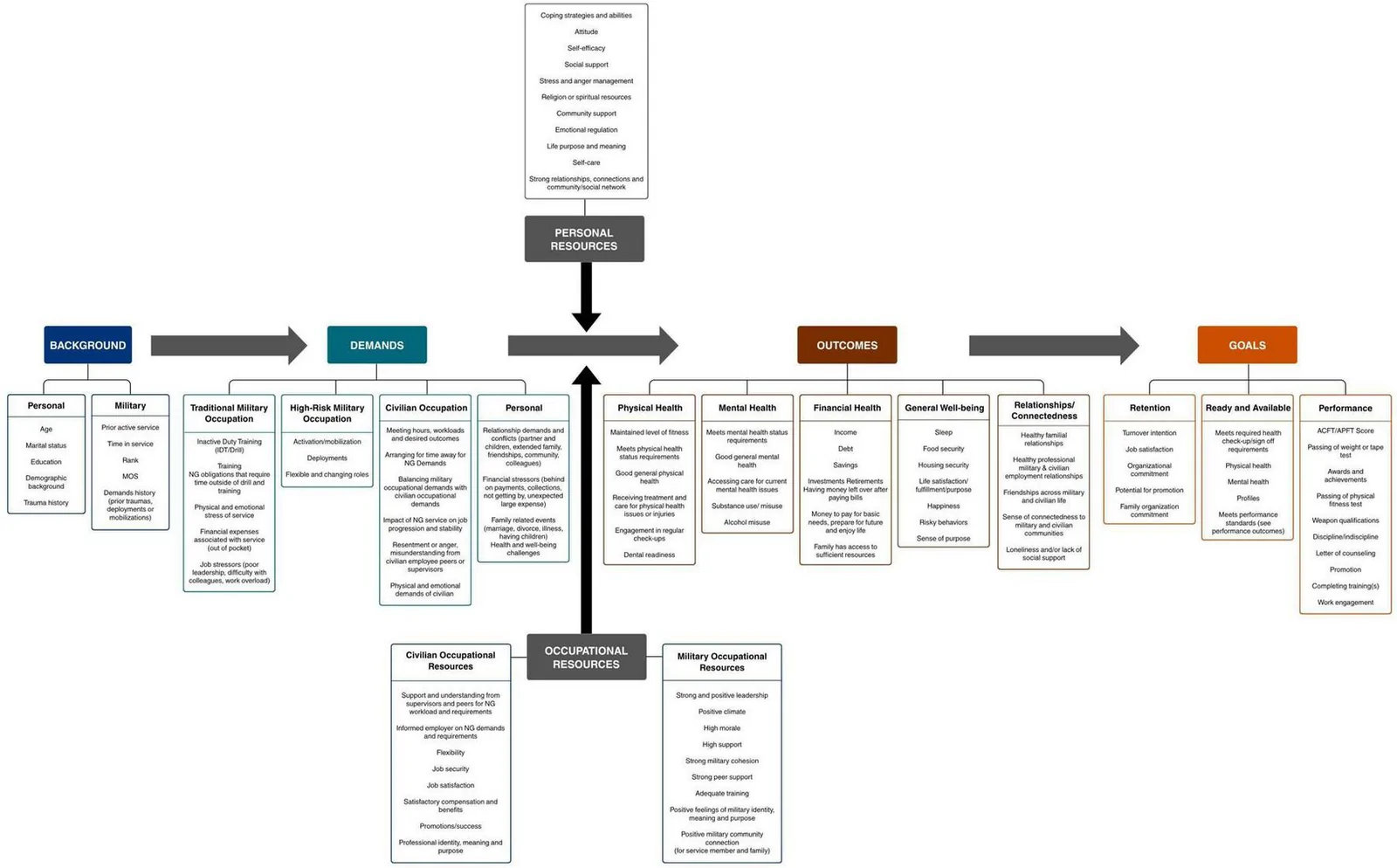
National Guard Occupational Demands Model (NGODM) full model.

### Goals

The model outlines three high level goals essential to a mission-ready NG force (see Figure 3). These include retention, readiness and availability, and performance. The model positions these goals as important to not only the individual service member but to the larger military organization.

**FIGURE 3:**
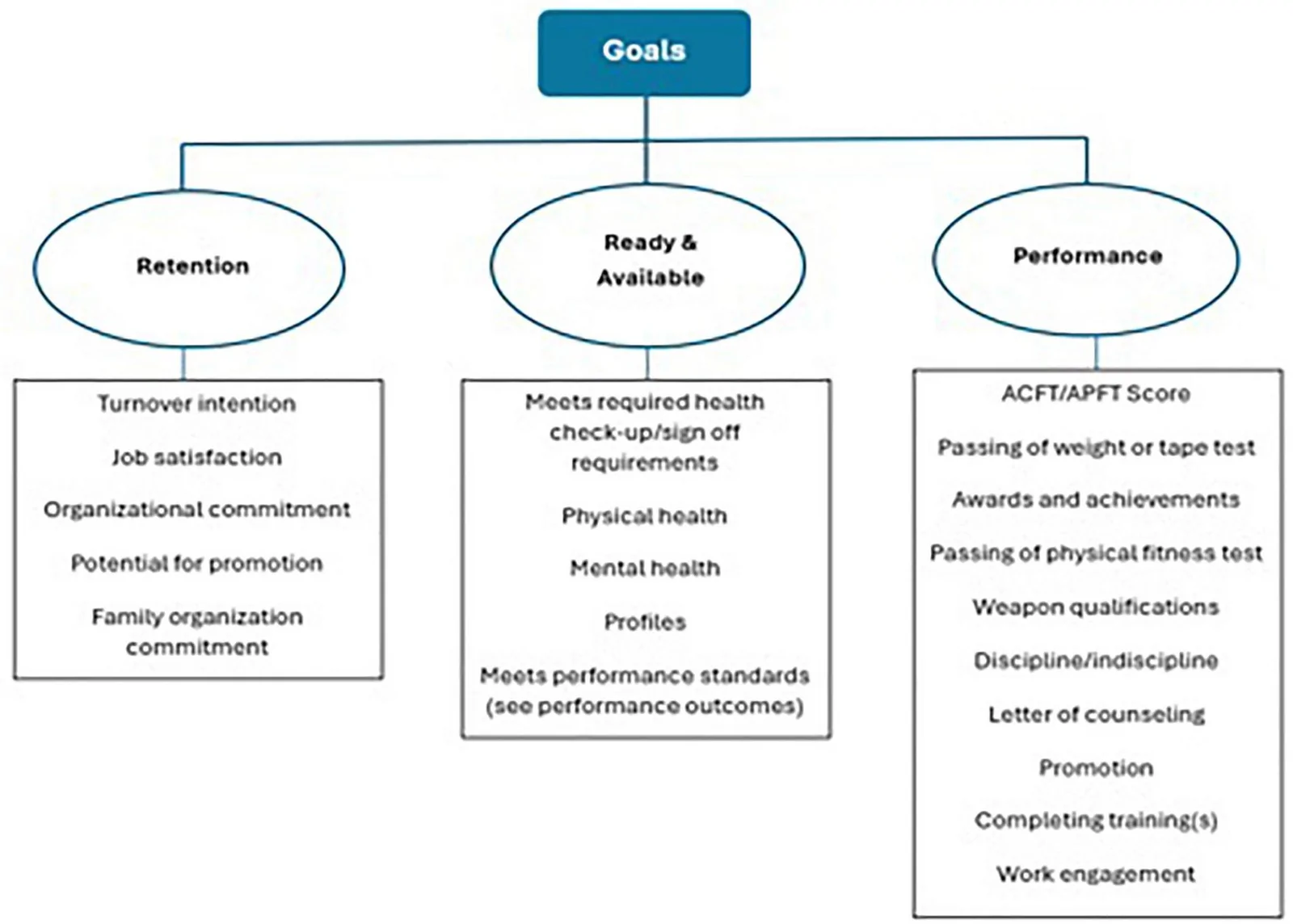
National Guard Occupational Demands Model (NGODM) goals.

#### Retention

A 2020 survey of approximately 15,000 NG members and reservists indicated that 65 percent of Army National Guard (ARNG) members and 79 percent of Air National Guard (ANG) members were likely to continue serving after their current obligation expired (Office of People Analytics, 2021). Retaining NG members preserves and prolongs the value of the significant investments associated with their recruitment, training, and integration. Improving retention is thus critical to maintaining a mission-ready force.

The NGOD model identifies several factors that may be used to measure or indicate retention. These include whether an NG member plans to continue with their service (turnover intention), satisfaction with service (job satisfaction), commitment to the NG (organizational commitment), opportunities to advance their career (promotion potential), and how their family feels about continued NG service (family organization commitment).

Several factors outlined within the proposed model have been shown to predict retention intention. The Personal Choice Model of military retention suggests that perceived support from leadership and peers is one of the strongest determinants of an individual’s intention to continue serving (Tidwell and Lucier-Greer, 2024). Units that foster open communication, mutual respect, and a culture of dependability among members are more likely to retain experienced personnel. Units that demonstrate these qualities are characterized by higher morale, a key variable linked to retention that reflects a service member’s enthusiasm, pride, and motivation to contribute to mission success (Ivey et al., 2015).

Rank and years of service also correlate closely with reenlistment likelihood, with junior ranks being less likely to continue with service, and intention to remain in the military increasing as service members move up the ranks (Office of People Analytics, 2021). This data suggests that opportunities for advancement and recognition may play a significant role in sustaining commitment. Additionally, the ability to balance military obligations with personal and family responsibilities remains an important determinant of retention. Members who experience excessive work-family conflict are more likely to consider separation, while those with strong family and organizational support tend to remain in service longer (Sims et al., 2024). Demographically, younger NG members are less likely to be retained, and women tend to expect and plan for a shorter military career than their male counterparts, due in part to the greater challenges they face in balancing their career and family role expectations (Vasterling et al., 2015; Smith and Rosenstein, 2017). As retaining trained and experienced personnel reduces future recruitment and training costs and increases readiness through sustainment and transmission of institutional knowledge, understanding and addressing retention is essential to maintaining a capable and mission ready NG force.

#### Ready and available

The NGDOM conceptualizes readiness and availability as the ability of the NG to be deployed, activated, or mobilized at both the federal and state levels. A strong, healthy, and fully deployable NG force is essential to maintain overall readiness and ensuring the organization can respond effectively to missions ranging from domestic emergency response to overseas operations. Between 2001 and 2019, more than 900,000 Guardsmen were mobilized for deployments, accounting for approximately one-third of the 3 million military personnel deployed during that period (Congressional Research Service, 2020). This operational demand underscores the importance of sustaining a force that is consistently prepared to mobilize when called upon. Readiness requires that members are medically, physically, and psychologically fit for duty, with completed health assessments and a current signoff verifying compliance with deployment standards (Department of the Army, 2018).

Unlike active-duty members who are provided guaranteed health care through the military health care system, NG members often face unclear, fragmented, and discontinuous care through TRICARE Reserve Select (TRS) coverage, which changes as their duty status changes. Many members have expressed concerns about gaps in coverage and administrative complications resulting from these transitions, which impede both medical and physical readiness (National Guard Bureau, 2022). As a result, continuity of care becomes a significant challenge for NG members and their families, especially when managing chronic health conditions or maintaining long-term readiness. Additionally, NG members live across their respective states, and access to TRICARE coverage can be limited.

A critical component of readiness involves reducing the number and duration of medical profiles, which are official records documenting a member’s temporary or permanent physical or psychological limitations. Extended profiles restrict training participation and reduce a unit’s deployable strength. Therefore, readiness goals should include early identification of injuries or mental or behavioral health issues, efficient treatment and rehabilitation, and timely return to duty. Educating members about injury prevention, physical conditioning, and mental wellness promotes a proactive readiness culture that emphasizes long-term health maintenance. Such education can also address issues of stigma surrounding accessing care for physical or mental health injuries. Health promotion leadership has also been shown to play an important role in encouraging wellness (Adler et al., 2014). By reducing the frequency and duration that NG members are nondeployable due to medical or psychological injury and ensuring accurate health readiness data, the NG strengthens its ability to mobilize rapidly and sustain mission operations at both state and federal levels.

#### Performance

Performance within the NG directly influences mission success, unit cohesion, and operational safety. The military’s emphasis on fostering a high-performance culture underscores the expectation that NG members consistently meet or exceed standards in physical fitness, weapons qualification, professional conduct, and technical proficiency (Knight, 2023). High-performing units execute missions more effectively, maintain stronger morale, and demonstrate superior adaptability in dynamic operational environments. Members who meet performance expectations enhance their own confidence, strengthen professional identity, and contribute to the collective trust essential for team effectiveness. Conversely, members who fail to meet performance requirements may face retraining or separation, both of which impact retention and readiness. Building a culture that values continuous improvement, accountability, and professional development embeds excellence as a shared norm.

At the same time, the unique dual demands of NG service, which include balancing civilian employment, family responsibilities, and military obligations, can create stressors that impair performance over time. Research indicates that chronic stress, fatigue, and inadequate recovery can reduce physical and cognitive functioning, increasing the risk of errors, injuries, and degraded mission performance (Johnson, 2025). Sustaining high performance requires a comprehensive approach that integrates physical training, mental health support, and effective leadership engagement. Programs that promote resilience, mentorship, and flexible scheduling can help maintain optimal performance while reducing stress. By investing in these performance enhancement systems, the NG not only strengthens mission effectiveness but also preserves the wellbeing and longevity of its members.

### Outcomes

The model outlines five outcomes that represent optimal wellness domains for NG service members (see Figure 4). These outcomes include physical health, mental health, financial health, general wellbeing and relationships and connectedness These NG member outcomes, are influenced by background characteristics, occupational demands, and the intervening constructs of personal and occupational resources. These individual outcomes are predicted to directly affect the organizational goals of retention, readiness, and performance.

**FIGURE 4:**
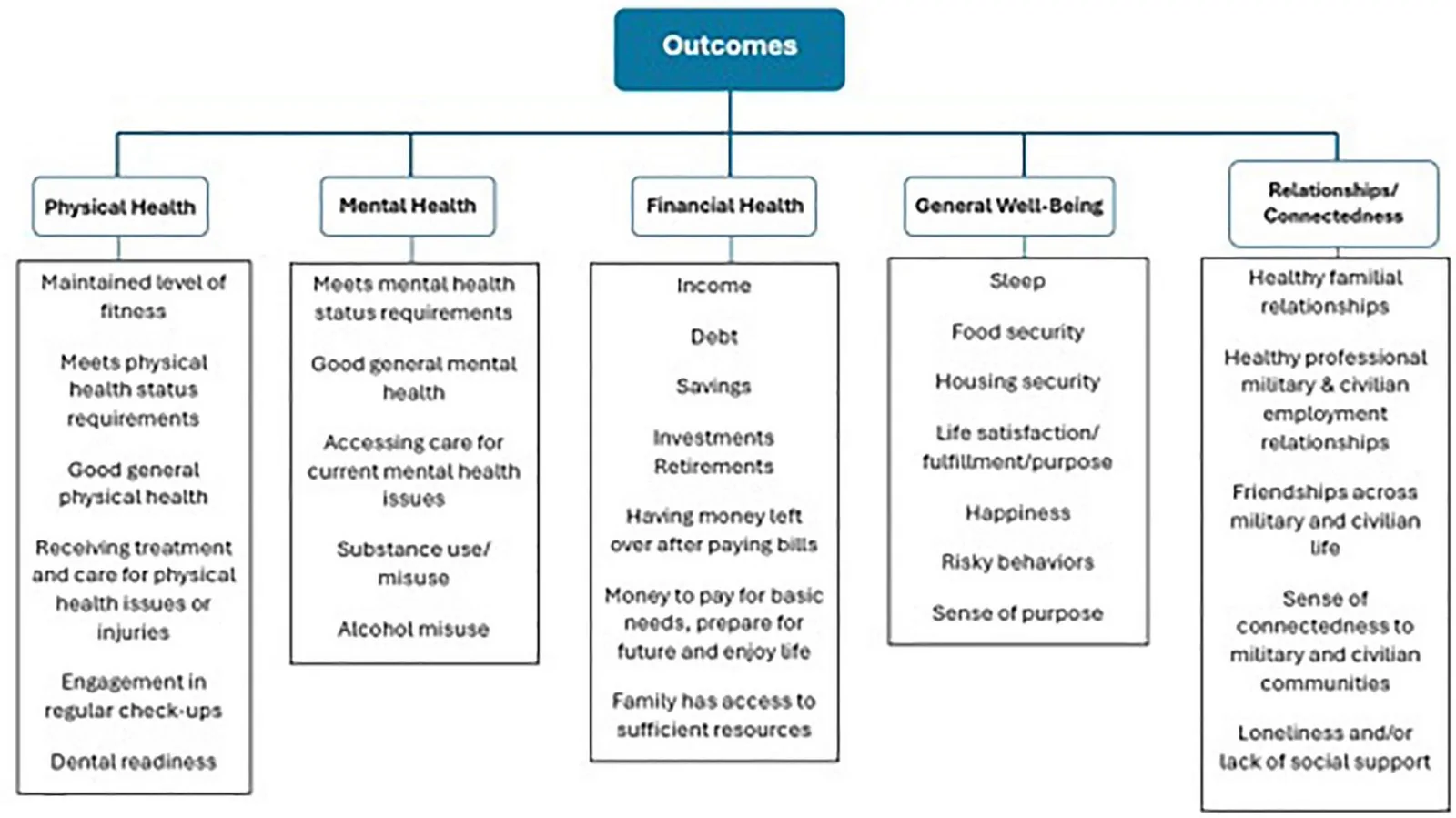
National Guard Occupational Demands Model (NGODM) outcomes.

#### Physical health

Physical health in the NG context encompasses both ongoing behaviors that sustain optimal physiological functioning and the resultant state of functioning itself. Physical health thus reflects a dynamic process in which sustained engagement in health-promoting behaviors, such as regular physical training, adequate sleep, injury prevention, and accessing preventive and acute medical care, produce measurable outcomes in strength, endurance, resilience, and overall fitness. In this sense, physical health reflects the extent to which service members maintain readiness and physical integrity over time. Maintaining a high level of fitness is central to ensuring readiness and the ability to deploy or mobilize effectively and. To this end, NG members must meet specific physical health status requirements through periodic assessments that evaluate overall fitness, endurance, and strength. Good general physical health not only supports performance but also contributes to confidence and supports identity as a capable service member. However, challenges such as musculoskeletal injuries, chronic pain, sleep disturbances, and weight management issues remain common and can affect both individual functioning and unit readiness (Adler and Castro, 2013; Hansen et al., 2018; Lincoln et al., 2018).

To sustain physical readiness, NG members are expected to receive regular medical check-ups and maintain compliance with health standards, including addressing blood pressure, cholesterol, and other indicators of general health. Access to appropriate treatment and care for injuries or chronic conditions is also vital, as unaddressed problems can lead to extended medical profiles that negatively impact individual deployability and unit readiness. Broader systemic issues such as obesity and poor sleep quality have been identified as readiness threats within Guard and Reserve components (American Security Project, 2025; Good et al., 2020). Consequently, engagement in regular check-ups, proactive injury management, and adherence to treatment recommendations serve as critical mechanisms for preserving operational capability and promoting long-term physical wellbeing within the NG.

#### Mental health

Mental health reflects the constellation of emotional, cognitive, and behavioral functioning that enables NG members to manage stress, maintain resilience, and perform their duties effectively; this requires both ease of access and willingness to seek care for routine mental health support and acute challenges. In the modern era, repeated deployments and increased domestic mobilizations, including sustained exposure to dangerous and uncertain conditions like wildfire management and civil unrest, have placed significant psychological burdens on NG service members, who must also contend with civilian stressors like economic uncertainty and a rapidly changing employment landscape. NG members regularly experience both routine operational stressors and sustained absences from civilian jobs and family support, in addition to potential exposure to trauma and loss. As a result, NG members often face distinct mental health challenges compared to active-duty personnel, as they return to civilian environments lacking the embedded support structures of full-time military life. These challenges can include posttraumatic stress disorder (PTSD), depression, anxiety, moral injury, anger dysregulation, and suicidality (Milliken et al., 2007; Britt et al., 2021). The variable tempo of NG service, compounded by barriers to military behavioral health resources and the pressure of meeting both military and civilian occupational obligations, increases vulnerability to untreated or underdiagnosed mental health conditions. Consequently, promoting adaptive mental health outcomes within the NG context requires addressing stigma, improving continuity of care, and fostering leadership and peer environments that normalize help-seeking and psychological readiness.

#### Financial health

Financial health is a critical determinant of NG members overall readiness and wellbeing. Financial health encompasses the adequacy and stability of an NG member’s economic circumstances, including employment, income, debt, savings, retirement funds, investment accounts, and ability to meet personal and family financial obligations. Financial stress is consistently cited as a primary source of strain among NG members and contributes to overall stress burden and its downstream psychosocial impacts (Bryan and Bryan, 2019). Having adequate civilian employment is likely a strong protective factor for NG members’ financial security. However, because they balance civilian employment with military responsibilities, NG members face risks of unemployment, underemployment and missed advancement opportunities due to training or mobilization (Hardison and Ginsberg, 2024). Many also incur unreimbursed expenses related to travel, equipment, or childcare during training periods, which can exacerbate financial strain (U.S. Government Accountability Office, 2017). Pay discrepancies between civilian and military wages contribute to economic insecurity, particularly for lower-ranking service members. Financial distress not only affects overall wellbeing but is associated with poorer health-promoting behaviors and lower perceived control (Elbogen et al., 2024). Addressing financial health within the NG population is therefore essential not only for individual welfare but also for retention and mission readiness.

#### General wellbeing

General wellbeing extends beyond the satisfaction of basic material needs and physical and mental health to encompass a gestalt sense of satisfaction, functioning, and quality of life. In the NG context, wellbeing can reflect the capacity to balance multiple life domains, including civilian employment, military service, family responsibilities, and community engagement, while maintaining a sense of personal meaning, purpose, and control. Factors influencing general wellbeing include not only stress management and emotion regulation, but also leisure, fulfillment, and happiness. On the other hand, dissatisfaction with life in the context of overwhelming stress burden can lead to risky behaviors and maladaptive coping. For example, substance use, particularly alcohol misuse, has been identified as both a coping strategy and a risk factor within military populations linked to deployment stress, cultural views around drinking for stress relief, and barriers to mental health treatment (Thomas et al., 2010). Improving general wellbeing within the NG context requires a comprehensive approach that addresses these interacting domains and supports NG members as citizen soldiers navigating complex and sometimes competing demands.

#### Relationships and connectedness

Relationships and connectedness refer to the quality and strength of interpersonal and community ties that foster belonging, mutual support, and social identity. For NG members, these constructs span both military and civilian spheres, each of which may offer, or fail to offer, vital sources of connection. Because NG members serve part time and often live geographically dispersed from their units, they may experience limited camaraderie or feel disconnected from the broader military culture (Rugo et al., 2020). Simultaneously, reintegration into civilian communities following deployment can be challenging, particularly when those communities lack understanding of military experiences. Strained family relationships, marital conflict, loneliness, or social isolation can further undermine resilience; experiences of major social disconnection, like divorce and separation from the military, are consistently linked to high risk for poor mental, social, and behavioral health outcomes like homelessness and suicide (Congressional Research Service, 2019; Kintzle et al., 2023; Vest et al., 2017). Conversely, supportive family systems, unit cohesion, and peer networks serve as powerful protective factors that buffer the effects of occupational stressors and promote retention (Rugo et al., 2020). Strengthening connectedness across these contexts is therefore central to sustaining both individual wellbeing and organizational functioning within the NG.

### Demands

Evidence shows that job demands are important causes for worker burnout, fatigue and various mental and physical health issues (Schaufeli and Taris, 2014). Job demands can be defined as the “physical, social, or organizational aspects of the job that require sustained physical or mental effort and are therefore associated with certain physiological and psychological costs” (Demerouti et al., 2001, p. 501). Additionally, Castro and Adler (1999) have argued that in the military context, OPTEMPO, which characterizes the frequency and duration of operational deployments, activations or rotations, is an important aspect of job demands that can impact on health, wellbeing, and readiness.

The importance of occupational demands within the military has long been understood as a key contributor to service member outcomes (Bliese and Castro, 2000). Yet to date the study of military demands has been primarily limited to active-duty personnel, or to the demands such as deployment and combat when NG members have been activated (Hardison and Ginsberg, 2024). Here we focus on both aspects of the job demands of the NG, including demands that mirror those of the active-duty component and those unique to the NG. In our model, occupational demands comprise four major categories: traditional military demands; high-risk military occupational demands; civilian occupational demands; and personal demands (see Figure 5).

**FIGURE 5:**
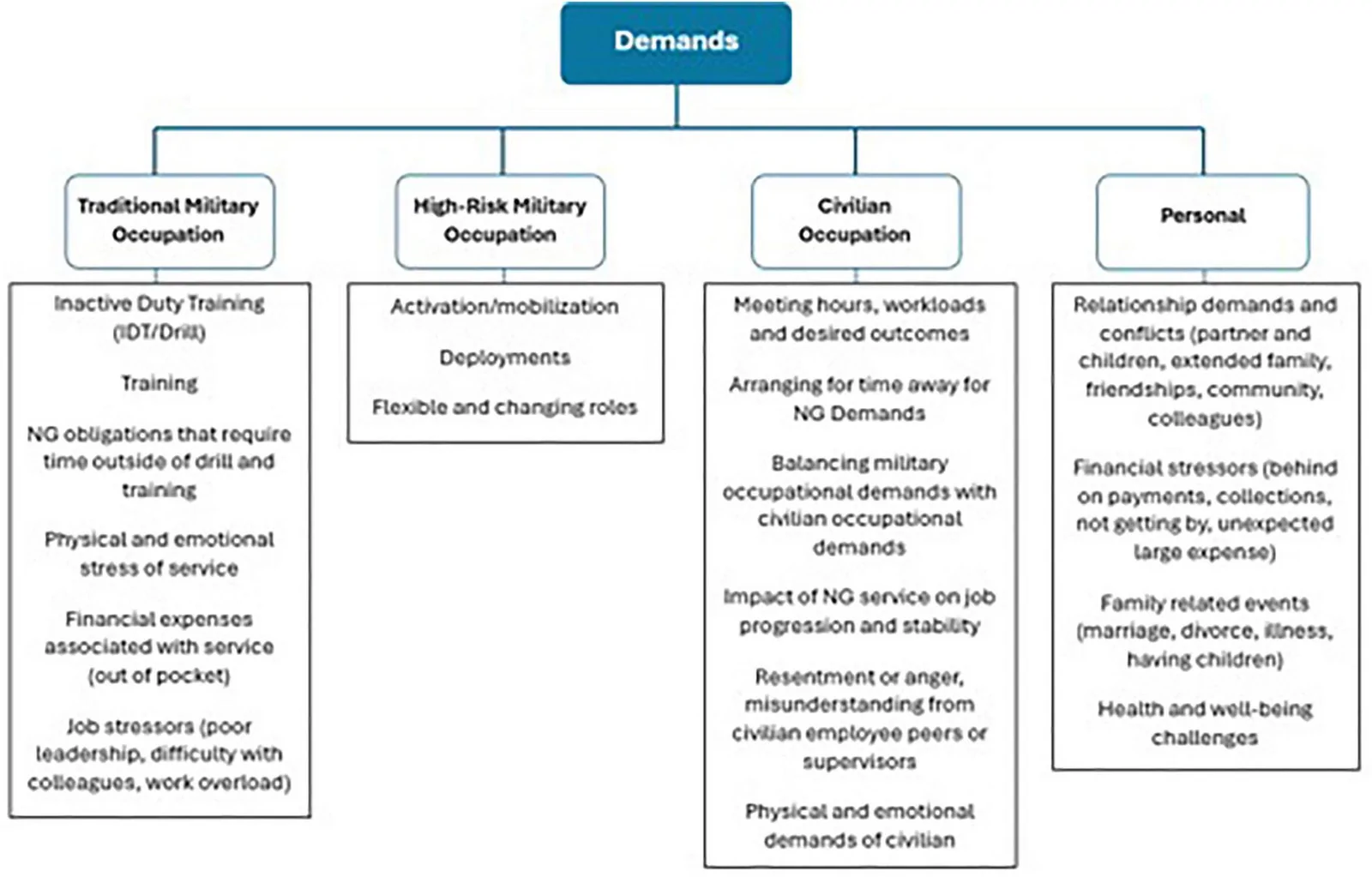
National Guard Occupational Demands Model (NGODM) demands.

#### Traditional military occupational demands

National Guard job demands are often incorrectly defined as limited to the absolute minimum of 1 weekend a month for drill and 2 weeks of annual training at a nearby active duty or NG training site. This view incorrectly depicts the NG as a relatively low-level, part-time commitment with very few demands, ignoring the traditional military occupational demands that NG members must manage, including mandated individual training, combat and unit training, required attendance at military schools for promotion, managing military work stressors (i.e., leadership challenges, work overload, issues with colleagues) among many others. In addition to these demands, NG members must also be prepared to quickly mobilize or deploy if called upon. This means they must also meet all deployment readiness standards, including health and dental assessments, legal and financial checks, as well as family-related issues. Many of these mandated military trainings require NG members to complete them outside of their typical weekend drills.

National Guard members also face the typical workplace challenges that active-duty personnel face, including work overload, task ambiguity, and uncertain training schedules. Conflicts with unit members also occur among NG units. On top of these “normal” military demands, many NG members are also required to travel long distances to attend weekend drill, often resulting in them having to pay out of pocket for travel and lodging costs, as NG do not receive per diem for attending drill.

#### High risk military occupational demands

During the wars in Iraq and Afghanistan, NG members deployed at a rate not seen since WW II, performing security duties in Germany and Italy that involved protecting U.S. bases, leading the peacekeeping missions in Kosovo and Bosnia and Herzegovina, as well as conducting combat operations in Iraq and Afghanistan (Defense Science Board Task Force, 2007). Despite the winding down of many of these conflicts, the tempo of overseas deployments for NG has not diminished. In addition to supporting the missions of the active duty, the NG also conducts missions that support their home states as well as other federal agencies. The NG are frequently called upon during times of natural disaster such as during weather events, forest fires or civil unrest, where they perform a variety of duties including civil policing, pandemics, security duties, fire suppression/mitigation tasks, assisting federal agencies, border patrols and drug interdiction operations. Such federal and state activations have increased in frequency and duration in recent years. Many of these roles are not part of NG traditional military training; thus, these tasks require additional training, which are most often conducted after they have been activated. The recent perceived politicization of certain types of federal and state mobilizations may also add to the stress of such high-risk demands.

#### Civilian occupational demands

As previously described, one of the distinguishing factors of NG service is that NG members most often have full-time civilian employment. This means they have a primary civilian employer for whom they must manage separate and additional demands. These can include managing hours and workload, meeting performance expectations and deadlines, navigating relationships with supervisors and colleagues, and the regular stressors associated with civilian employment. However, in addition to these traditional employment demands, NG must make time for their service demands. They must often take time away from their civilian employment and may have to deal with related challenges from colleagues or supervisors despite legal protections meant to protect their civilian employment status (Reynolds et al., 2019).

Serving in the NG is viewed by many as a part-time job that occurs alongside a civilian job. Inevitably there will be conflicts between the NG member’s civilian job and their service-related demands. Naturally, the NG member’s civilian employer expects them to prioritize their full-time civilian job over their part-time military one, while the military expects the civilian employer to acquiesce to the needs to the NG (Hardison and Ginsberg, 2024). This conflict can place the NG member in the difficult position of having to navigate between critically important commitments. The extent of the conflict or competing demands between civilian and NG employment will depend on the nature of the civilian job and the flexibility of the civilian employer, with the military typically being the most inflexible. At the same time, having supportive civilian employment is a protective factor in the context of increased military demands. Thus, it is critical that civilian employers appreciate and accommodate NG members in their role supporting the Nation, the State, and their communities.

#### Personal demands

Along with occupational demands, all individuals have demands related to their personal life. These demands might include meeting basic needs, managing relationships, life stressors such as major events or illnesses, providing for a family, or personal conflicts. Personal demands also describe dealing with challenges to the major model outcomes, such as mental and physical health issues, financial challenges, substance use and general wellbeing (Chen and Fellenz, 2020). Military service in any form is demanding on the physical body and can often create emotional health challenges; combat roles, where NG members have served and continue to serve, are particularly taxing. In addition, nearly a third of NG members have prior military service (Military Leadership Diversity Commission, 2011) - thus it is likely that many may still suffer from physical and psychological injuries they acquired while on active-duty, although at the time of joining the NG they may not have been fully aware of the extent of these active-duty related injuries. NG members may also become injured while performing their civilian job, or existing injuries may become exacerbated by their NG service.

All NG members must meet the same level of physical and psychological readiness as active-duty personnel yet have considerably less support than the active component (Blow et al., 2011; Pinna et al., 2017; Schuh et al., 2016; Sutton, 2026). NG members who become physically or psychologically unable to perform their duties may be separated from the NG. This can lead to NG members overlooking and undermining their injuries and conditions, ultimately not getting the care they need.

### Occupational and personal resources

Occupational and personal resources describe variables that may moderate the impact of demands on outcomes and goals (see Figures 6, 7). These are factors that may mitigate the risk of occupational demands and are expected to influence the relationship between demands and outcomes. Resource variables operate by providing protection against the negative effects of demands while also having a potential direct effect of NG outcomes and organizational goals. These variables may also be important when developing interventions, training or policies that aim to build resilience or treat challenges associated with occupational stress.

**FIGURE 6:**
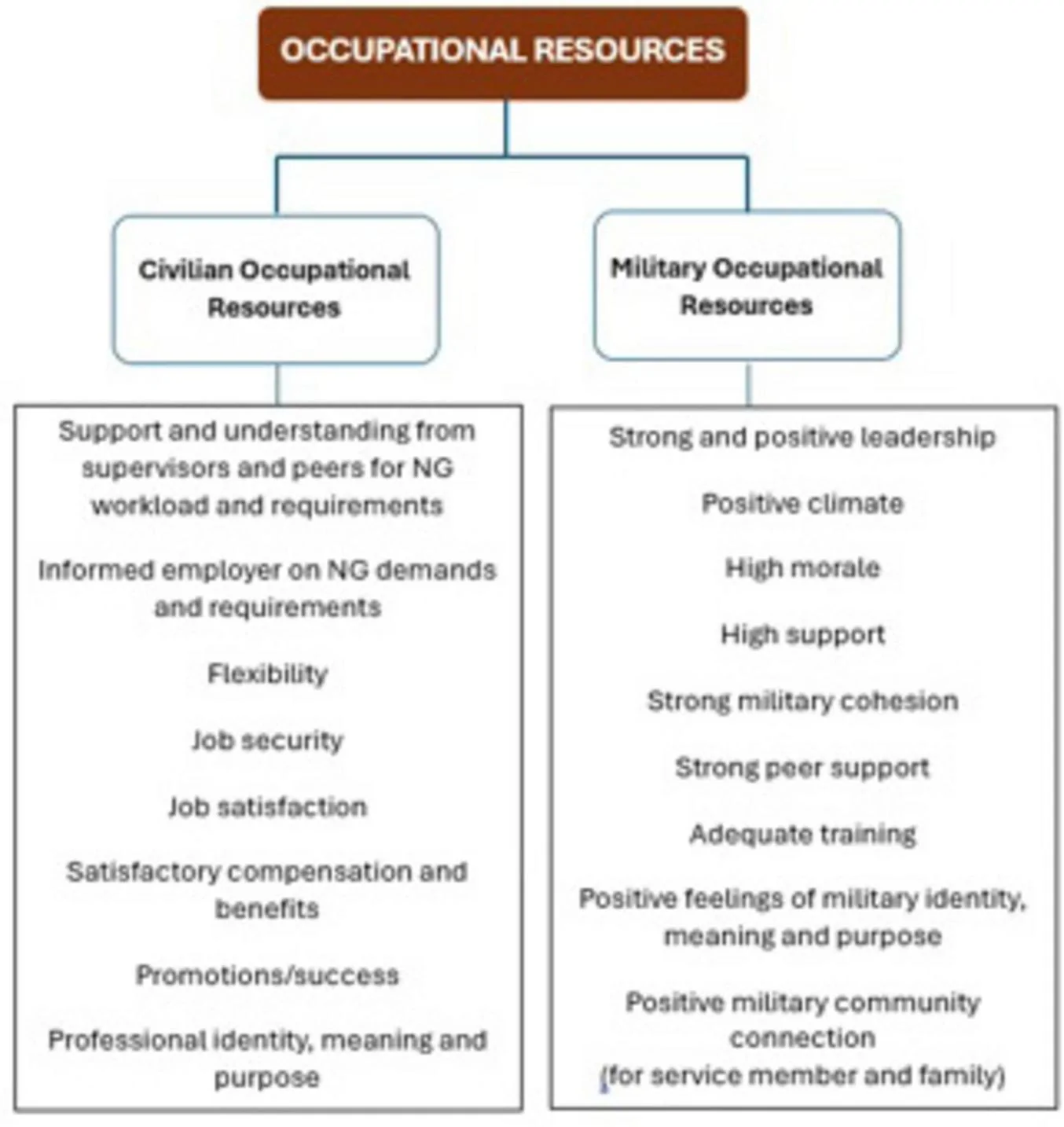
National Guard Occupational Demands Model (NGODM) occupational resources.

**FIGURE 7:**
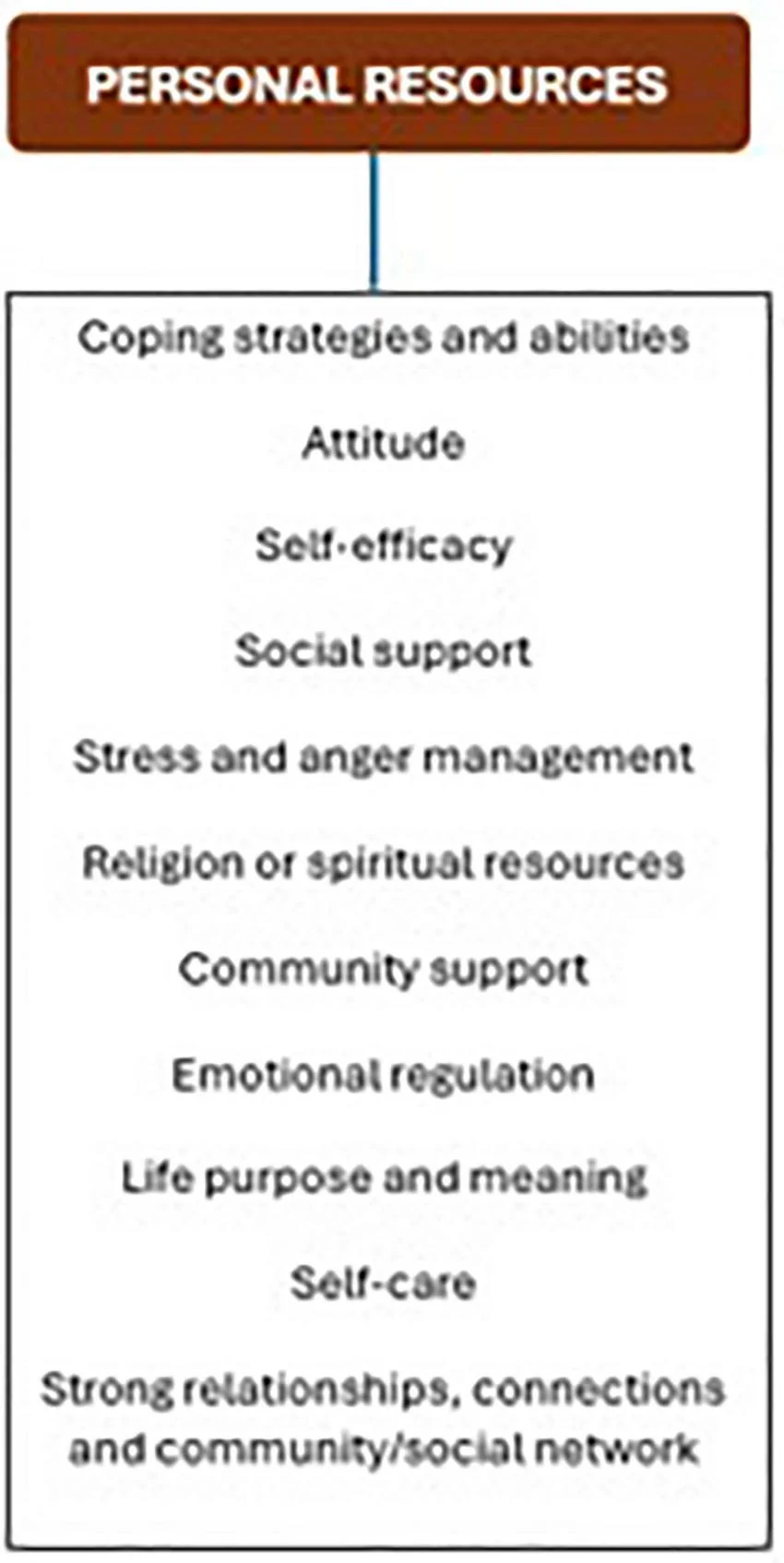
National Guard Occupational Demands Model (NGODM) personal resources.

#### Civilian occupational resources

NG members most often balance both civilian and military careers. Military obligations offer almost no flexibility, meaning civilian employment often takes the brunt of the impact of managing these dual roles. Flexible, understanding and supportive civilian career environments may be a protective factor in reducing the stress and impact caused by these competing occupational demands (Kirchner and Herd, 2021). In both research findings and anecdotal reports, NG members describe challenges with their civilian jobs. These include lack of opportunity for career advancement, peers and supervisors who are unsupportive of NG commitments, concern of losing their job, roadblocks to accessing civilian jobs, low pay or benefits, reluctant employers, and loss of job due to NG obligations despite federal law protections (Kinney, 2025; Lewis, 2026; NPR, 2015; Werber et al., 2024). These challenges often mean the military may be the only source of employment at times for NG members. Because employment is a protective factor for many aspects of wellbeing (Gedikli et al., 2022), having a supportive, enjoyable and stable civilian job may be an important protective factor for model outcomes and goals.

#### Military occupational resources

Military occupational resources have been well-defined and robustly investigated in the active-duty population. It is likely many of these resources will map onto the NG context, although most have not been tested explicitly within the NG. Examples include morale (Bogdan and Balica, 2024), cohesion (Campbell-Sills et al., 2022), the development of a professional military identity (Heward et al., 2024), training (Zueger et al., 2023), peer support (Hom et al., 2017), feelings of meaning and purpose around service (Bauer et al., 2018), and a strong military community. Consistent with findings with active-duty populations, leadership is an essential occupational resource (Adler et al., 2014) and likely one of the most impactful model variables. To highlight the crucial, pervasive influence of leadership on service member wellbeing, investigators have delineated domain specific leadership characteristics, including Combat Operational Stress Control leadership; Health promoting leadership; Resilience training leadership; Emotion regulation leadership; Post-traumatic growth leadership; Sleep leadership; Moral leadership; Ethical leadership; and Family supportive leadership (Adler et al., 2014). It is important to note that impactful leadership within a part-time force may look different than in the active-duty force. Adapting domain specific leadership to the NG, as well as the other military occupational resources, and researching their role in promoting positive outcomes and goals could be essential in developing stress mitigating interventions for NG personnel.

#### Personal resources

Personal resources describe individual attributes or abilities that may assist in mitigating the negative impacts of stress and demands. Examples of these include self-efficacy, stress and anger management skills, emotional regulation and effective coping strategies. Personal resources may also include different types of social support, such as a strong community, social connections, or religious or spiritual resources. Access to healthcare is also an important resource that will most likely impact how military demands affect outcomes and goals. Lastly, individuals’ ability to engage in self-care as well as strong feelings of purpose and meaning, may also be personal resources that protect against stress and demands.

### Background

These variables identify background, demographic and military occupational factors that may impact the relationship between occupational demands and outcomes (see Figure 8).

**FIGURE 8:**
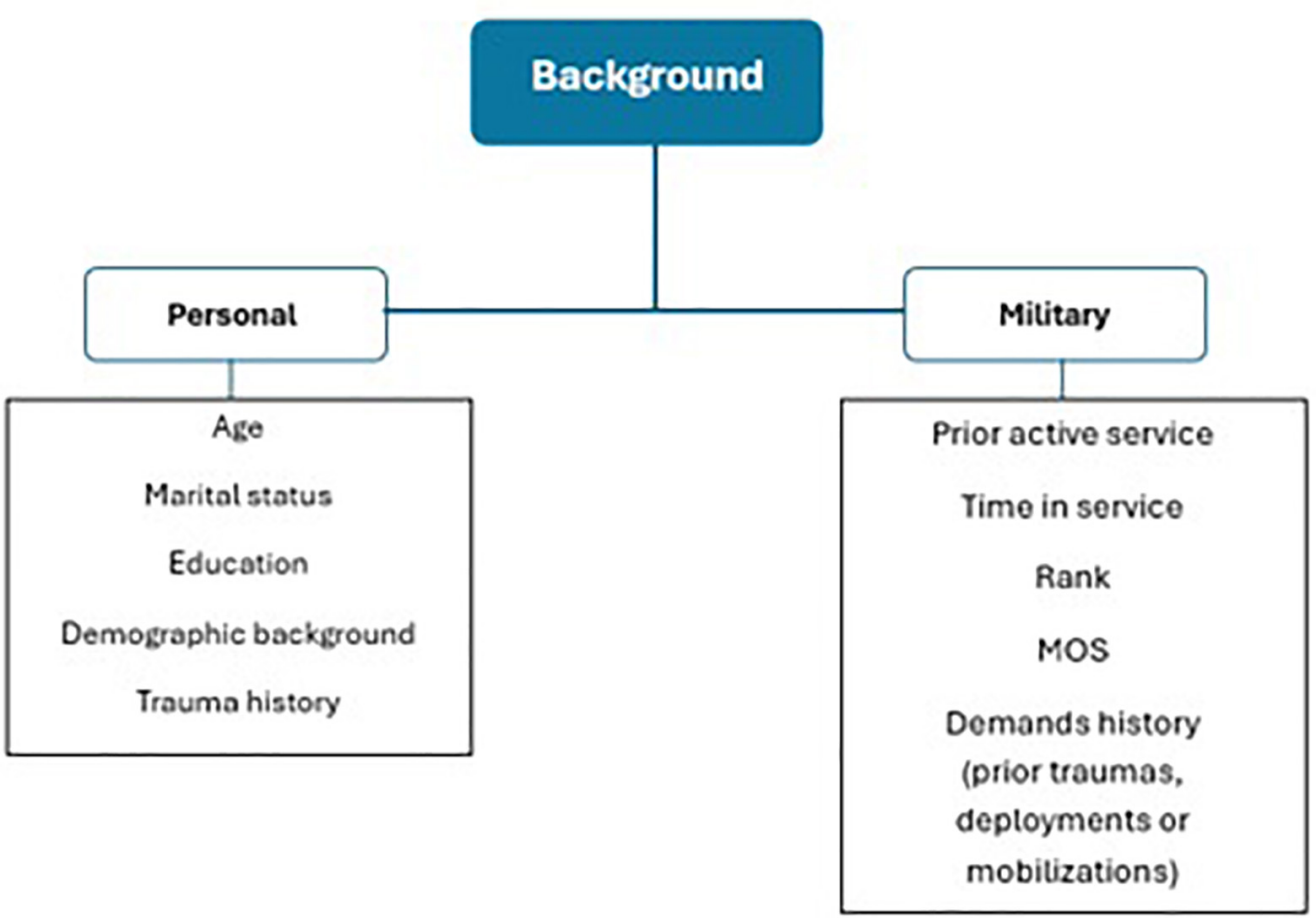
National Guard Occupational Demands Model (NGODM) background.

#### Personal background

Demographic variables such as age, marital status, education, sex or gender, race and ethnicity, sexual orientation, and religion are all examples of personal background factors that may impact how service members experience demands. Further, individuals enter the NG with a personal history and a pre-existing level of wellbeing that may impact their experience of serving. A personal trauma history may make individuals susceptible to experiencing additional trauma or vulnerable to the development of PTSD (Ozer et al., 2003; Beliveau et al., 2019). Conversely, a trauma history may also facilitate the development of adaptive coping or resilience in the face of stressors (Angel, 2016; Tsai et al., 2015). Personal background and demographic characteristics are important to consider when examining the impact of service stressors.

#### Military background

Approximately 31 percent of individuals who serve in the NG have previous active-duty military experience (Military Leadership Diversity Commission, 2011). Prior service may provide protection against NG stressors through experience dealing with military demands, creating resilience and positive coping mechanisms that can be applied to their NG service (Wilk et al., 2020). Having a history of service may also create susceptibility to the demands of NG, particularly if the demands of prior service lead to negative outcomes such as mental or physical health issues, anger or frustration toward leadership, or maladaptive coping mechanisms (Xue et al., 2015). In short, it is possible both the positive and negative experiences of prior active-duty service will impact outcomes during NG experiences. Understanding what NG members bring to their service, such as a history of deployments, combat experiences, health issues, substance abuse issues, as well as factors such as perception of leadership and reasons for leaving active duty, may all be important in examining the impact of NG demands on outcomes and goals.

## Strengthening NG research, intervention and policy through the NGODM

The NGODM addresses a persistent gap in the NG wellbeing literature and advances the three aims set out in this paper. First, it synthesizes a fragmented empirical literature on NG stressors and domains of wellbeing into a coherent set of constructs encompassing service demands, resources, individual outcomes and organizational goals. Second, it extends the Military Occupational Mental Health Model (MOHM; Adler and Castro, 2013) beyond the active-duty context on which that model was built, explicitly representing the dual civilian-military role and unique structural demands that characterize NG service. Third, it integrates these constructs and their proposed associations into a theoretical model from which testable propositions related to NG service demands and risk and protective factors impacting outcomes can be derived, providing a foundation for research, clinical intervention and policy aimed at supporting NG service members.

While the NGODM provides many potential relationships and associations to be explored, the following represents a set of core theoretical propositions generated by the model. These propositions broadly describe the NGODM’s principal predictions.

(1). Understanding the dynamics of NG service is necessary for understanding how citizen soldiers experience impacts individual and family wellbeing outcomes and unit and organizational readiness.

(2). The unique demands of missions performed by NG directly influence individual wellbeing outcomes, leadership approaches, organizational goals, policies and programs.

(3). The distinct feature of NG service requiring the balancing of military and civilian demands also directly influences wellbeing outcomes, leadership approaches, organizational goals, policies and programs.

(4). Occupational and personal resources mitigate the impact of NG demands on individual outcomes and organizational goals.

(5). Consideration of the NG’s unique military culture is important in the development and implementation of approaches aimed at sustaining the readiness of NG personnel.

Within each of these core propositions, three classes follow from the model. The first concerns relationships that have been established in active-duty populations but have yet to be confirmed with NG service members, such as the positive relationship between military occupational resources including strong leadership, high morale and cohesion and outcomes and goals such as health, job satisfaction and turnover intention. The second concerns associations that may provide insight in all military populations, including the role of financial wellbeing in service member readiness, the association of identity or purpose with health outcomes, performance or retention, and whether social connections can lessen the negative impact of service demands on wellbeing. The third, and the clearest expression of the model’s extension of the MOHM, concerns relationships that are unique to NG service. This includes the role civilian occupational resources such as job satisfaction, job flexibility and supervisor support might play in protecting against the potential negative impacts of NG service stressors. It also includes testing how the unique demands of NG service, such as drill, balancing civilian and military occupations, and quick and frequent mobilizations, affect NG outcomes and goals, and what factors mitigate those relationships. Investigating these associations empirically can begin to address the gaps and limitations described within the NG literature and provide a foundation for the development of interventions or policies that may better support those who serve in the NG.

A key benefit of testing the proposed relationships in the NGODM is that results can directly lead to intervention or policy development that can strengthen support for NG. For example, should social connectedness or strong leadership be shown to mitigate the risk associated with NG military demands and increase positive outcomes or goals, interventions could be developed aimed at strengthening these areas. A previous example described the theoretical basis for hypothesizing a relationship between civilian occupational resources and outcomes and goals. Empirical investigation of these associations could facilitate development of training interventions for civilian employees or policies aimed at strengthening occupational resources. For a final example, the model describes the cost burden associated with NG service as distinct from those associated with active duty military service demands. Empirical investigation of these associations might yield insight into pathways from NG specific cost burden through lower financial wellbeing to retention or performance outcomes, informing policy changes around reimbursement for out-of-pocket service costs.

Several factors or constraints and limitations should be noted when considering the application of the NGODM to future NG related research questions. The model was developed through a narrative synthesis of the theoretical and empirical literature, informed by the authors’ clinical and research expertise with this population. This approach was appropriate to the aim of building an integrative framework where the underlying evidence is uneven and dispersed, but it carries the possibility that relevant work was not captured and that the selection and organization of constructs reflect the authors’ judgment. Additionally, similar to the MOMHM, this framework utilizes a multi-level perspective, where effects and interventions can occur at the individual/unit, leader and organizational level (Bliese and Castro, 2000). We organize analytic levels for model constructs broadly as follows. Individual-level constructs include personal and military background, personal demands and resources, and physical health, mental health, financial health, and general wellbeing outcomes. Retention intention, readiness, and performance can also be operationalized at the individual level, though in aggregate they represent organizational-level goals, highlighting how construct level depends on a particular study’s internal logic. Interpersonal constructs include family and peer support, relationships, social connections, and connectedness, across personal resource and outcome-level domains. Civilian-occupational constructs include workload, employment stability, job satisfaction, job flexibility, and supervisor and colleague support. Military unit/leadership constructs include training demands, work overload, task ambiguity, unit cohesion, morale, peer support, and domain-specific leadership. Organizational constructs include the aggregate goals of retention, readiness and availability, and performance, but could also include institutional policies that influence military occupational resources and demands, depending on a given study’s operationalization.

As previously stated, this model is proposed as a starting point for building knowledge that more adequately accounts for the unique and multidimensional stressors associated with NG service. Researchers are encouraged to test, adapt and advance the model to better represent the relationships, outcomes and goals presented. While the hypothesized links between model constructs are based on existing empirical knowledge, experience, and expertise, additional factors or variables may emerge based on new evidence; empirical testing may not support hypothesized associations; and model constructs may operate differently than initially proposed. In other words, we hope and expect that the model will evolve as evidence develops.

It is also important to note that this model represents the experience of part time NG service members. A minority of NG members are full-time personnel serving under the Active Guard Reserve program; their service demand experience is not fully represented by the NGODM. Lastly, NG family members are incredibly important to understand NG service members’ demand and support contexts. They are included in the model, but with regard to how they impact the serving Solider. NG families have their own unique demands and experience that have yet to be conceptualized or adequately studied. Future work should consider exploring how active-duty family models of wellbeing and support could be adapted to better serve NG families.

## Data Availability

The original contributions presented in this study are included in this article/supplementary material, further inquiries can be directed to the corresponding author.
